# Cutaneous leishmaniasis and iron metabolism: current insights and challenges

**DOI:** 10.3389/fimmu.2024.1488590

**Published:** 2024-12-03

**Authors:** Aicha Assouab, Ayyoub Kihel, Myriam Rouahi, Mathilde Larribau, Zoubida Karim, Khadija Akarid

**Affiliations:** ^1^ Health and Environment Laboratory, Hassan II University of Casablanca, Biochemistry, Biotechnology and Immunophysiopathology Research Team, Ain Chock Faculty of Sciences, Casablanca, Morocco; ^2^ University of Toulouse III, INFINITY, INSERM UMR1291, CNRS UMR5051, Toulouse, France

**Keywords:** iron, metabolism, hepcidin, *Leishmania*, cutaneous leishmaniasis

## Abstract

Leishmaniasis is a vector-borne parasitic infection induced by protozoa of the genus *Leishmania.* The disease spectrum ranges from skin lesions to visceral leishmaniasis, which is fatal if untreated. The cutaneous leishmaniasis is characterized by a clinical polymorphism of lesions with a broad range of severity ranging from a self-limited lesion to multiple disfiguring lesions stigmatizing the patient for life. Although iron is required for several process of *Leishmania* infection including survival, growth and virulence, the number of studies on host iron metabolism during this infection remains limited. Iron homeostasis in the body is finely regulated by hepcidin, a hyposideremic peptide highly expressed in the liver. In infectious contexts, hepcidin plays additionally an antimicrobial role, acting through various mechanisms such as retaining iron in tissues, modulating the immune response, and operating as a defensin against gram-negative bacteria. This review mainly summarizes the most important interconnections between iron metabolism, hepcidin and leishmaniasis. A deeper understanding of iron metabolism in this context could help in developing innovative treatment strategies that target the parasite while simultaneously reinforcing host defenses.

## Graphical Abstract

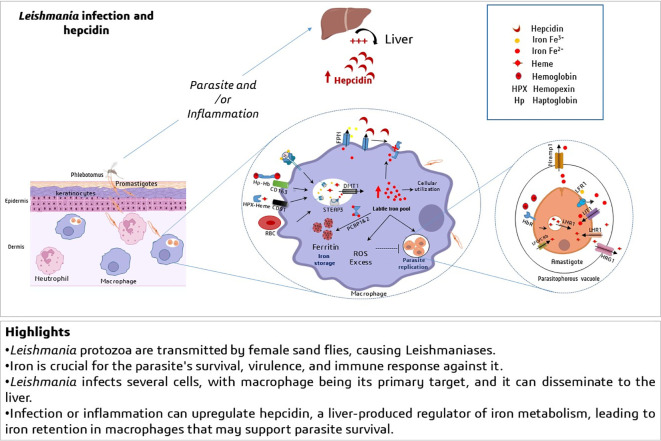

## 1 Introduction

Leishmaniasis is a vector-borne parasitic infection caused by a protozoan parasite belonging to *Leishmania* genus, transmitted by the bite of infected female phlebotomine sandflies. This infection manifests in three clinical forms that are cutaneous leishmaniasis (CL); mucocutaneous leishmaniasis (MCL), and visceral leishmaniasis (VL), affecting organs such as spleen and liver (1). Leishmaniasis is endemic in almost 100 countries across the globe, in tropical and subtropical region including Mediterranean basin. Worldwide, leishmaniasis has affected more than 12 million people, with an annual incidence estimated 0.7 to 1 million cases. Moreover, 350 million individuals are exposed to the risk of acquiring the infection (2–4). CL is the least severe and common form of leishmaniasis, manifesting as ulcers, ulcero-crusted lesions and nodular lesions on exposed parts of the human body, leaving disfiguring scars (1, 5). The species associated with CL involve *Leishmania (L) tropica*, *L. major, L. aethiopica*, and *L. infantum* in the Old World, notably Mediterranean Basin, the Middle East, the Horn of Africa, and the Indian subcontinent. Whereas, New World CL is endemic to South and Central America through the presence of *L. mexicana, L. amazonensis, and L. braziliensis* species (2). In North African countries including Morocco, CL is caused mainly by two species: *L. major* and *L. tropica*, and is still a public health issue (6, 7). In fact, *L. major* and *L. tropica* are associated with clinical polymorphism in terms of aspect, incubation period and recovery time. *L. major* lesions are multiple, acute healing in 4–6 months, whereas *L. tropica* lesions are mainly single, and last more than 1 year, confirming the chronic tendency of this form of CL (8, 9).

Iron is essential for most living organisms including *Leishmania* parasite (10). Iron is involved in many physiological processes via its redox state and electron transferring, which changes easily between Fe^2+^ and Fe^3+^. Iron is necessary for cellular metabolic activity, as it has optimal chemical properties for electron transfer, facilitating biochemical reactions including oxygen transport, ATP production and DNA synthesis. In the body, iron exists in two main forms: heme iron (porphyrin ring that bind to iron at its center) that is crucial for binding and release of oxygen by hemoglobin and myoglobin in the bloodstream and muscle tissues, respectively, and non-heme iron mostly bound to transferrin for transport into body cells (11). The main sources of iron are absorption through the diet in the duodenum, the release of recycled iron from macrophages and the release of stored iron within hepatocytes (12). Laranjeira-Silva et al. have published a comprehensive and detailed review discussing the importance of iron and heme acquisition, use and metabolize by *Leishmania* parasite. Briefly, as the parasite cannot synthesize heme, and lacks cytosolic iron storage proteins, *Leishmania* has developed strategies to acquire and use these nutrients, through the expression of specific transporters and enzymes that promote iron uptake and heme degradation. The authors described how *Leishmania* acquires heme, particularly via *Leishmania* Heme Response 1(LHR1) and heme transporter, the LFLVCRb. The iron uptake is mainly supported by *Leishmania* Iron Transporter 1(LIT1) and *Leishmania* Ferric Reductase 1(LFR1). Therefore, the regulation of iron and heme is meticulously orchestrated to ensure availability, when necessary. Detoxification of toxic heme and iron is controlled by the parasite by maintaining iron homeostasis and antioxidant system such as iron-dependent superoxide dismutases (SODs) [for review, see (13)].

## 2 Host Iron metabolism and *Leishmania* infection

Significant progress has been made in understanding iron transport in mammals and its regulation. Dietary Fe³⁺ is reduced to Fe²⁺ to be transported via a proton co-transporter named DMT1 (Divalent Metal Transporter 1 or SLC11A2) located in the brush border membrane of enterocytes (14). Iron is released into circulation at the basolateral pole via ferroportin (FPN or SLC40A1), the only iron exporter so far. The export of iron by FPN requires ferroxidase activity, allowing transferrin to bind circulating iron in the form of Fe³⁺ (15). In the blood, absorbed iron is quickly bound to transferrin (TF), an abundant binding protein with high affinity that can neutralize the reactivity of iron. Most cells in the body can assimilate iron bound to TF through the ubiquitous receptor TFR1, which has a high affinity for the iron-TF complex. Endocytosis mediated by TFR1 allows for the extraction of iron from TF in intracellular endosomes at acidic pH. The released Fe³⁺ is then reduced to Fe²⁺ by a reductase called steap3, and iron is released into the cytosol through DMT1 located in these endosomes (16, 17, 18). A small amount of heme iron can be found in circulation; hemopexin and haptoglobin are plasma proteins that bind heme and free hemoglobin, respectively, limiting their toxic effects. Both complexes are taken up by specific receptors (CD91 for the heme/hemopexin complex and CD163 for the hemoglobin/haptoglobin complex) expressed on the membrane of the spleen and liver macrophages, as well as in hepatocytes. These proteins play an important role in iron elimination in hemolytic diseases (19, 20).

Erythroid cells in the bone marrow are the largest consumers of iron. 20 to 30 mg are used each day to form hemoglobin in newly produced erythrocytes. Macrophages in the spleen and liver recover heme iron storage and recycling from aging erythrocytes after phagocytosis and catabolism of heme by heme-oxygenase (HO-1). Mobilizable iron is thus re-transferred to plasma transferrin for redistribution to tissues. FPN is essential for this iron export by macrophages (18). In all cells, unused iron is stored in a non-reactive form, thanks to ferritin (Ft) that stores up to 4,500 iron atoms (21). Fe²⁺ may be delivered to Ft by cytoplasmic chaperones such as PCBP1 (poly (rC)-binding protein 1) (22, 23). At high concentrations, iron-loaded Ft can be incorporated into lysosomes and crystallize. This leads to the formation of hemosiderin, which is not easily mobilizable (24).

Interestingly, macrophages are crucial target cells for *Leishmania* parasite to support its survival, growth and virulence. Prior to reaching macrophage, the parasite is subjected to interactions with diverse immune cells and subsequently escape them. In fact, the skin is the first line of physical and immune protection at the site of pathogen entry. After inoculation of *Leishmania* into the dermis, promastigotes interact with serum components, leading to activation of the complement system, which is responsible for the opsonization of around 90% of inoculated parasites. However, *Leishmania* has developed mechanisms to escape lysis. Furthermore, infection site comprises a vast population of resident cells such as keratinocytes (25). These later, are squamous epithelial cells that form a first line host barrier for the parasite and contribute to the immune response. *L. major* rapidly stimulates keratinocytes, which in response produce immunomodulatory factors (IL-12, IL-1β, osteopontin, IL-4 and IL-6) that influence the progression of the infection (26, 27). However, keratinocytes exhibit a high rate of iron acquisition and storage due to the constant renewal of the epidermal layers. Although it is not yet established whether the parasite utilizes this intracellular iron in keratinocytes, evidences suggest that local cutaneous inflammation significantly increases iron retention in the skin disrupting overall iron metabolism (28). This retained-iron likely plays a role in the early replication and survival of the parasite, from the onset of infection. Further studies must help to understand the parasite behavior in the initial phase of infection.

The polymorphonuclear neutrophils (PMNs) are the first phagocyte lineage recruited to infection site ensuring lytic and phagocytic functions against *Leishmania* parasite through the production of microbicidal factors ROS, nitric oxide (NO) and neutrophil extracellular traps (NETs). Nevertheless, the parasite possesses the capacity to manipulate neutrophils in its favor, thereby facilitating its dissemination. Indeed, *L. major* reduces the antimicrobial activity of neutrophils, thereby increasing the spread of the parasite when infected neutrophils are ingested by macrophages (29). The macrophages are the main host cells of *Leishmania* parasites, they influence the outcome of the infection by limiting or facilitating the growth of the parasites, which ultimately leads to the development or management of leishmaniasis. This duality is related to the pattern of differentiation of naïve macrophages towards M1 (classically activated) or M2 (alternatively activated). M1 macrophages are phagocytic cells known for their strong antimicrobial activity whereas M2 forms exhibit an anti-inflammatory and immunomodulatory phenotype creating an environment where *Leishmania* can evade the immune system and multiply (30). *Leishmania* parasites are found in phagolysosomal compounds such as a niche in macrophages, to proliferate as amastigotes. Therefore, the efficient transfer of iron biomolecules to these parasites can be classified into three distinct phases: the initial uptake of iron-containing molecules by macrophages and their subsequent translocation to the cytoplasm, then to the phagolysosomal compartment where *Leishmania* will assimilate iron (10). To oppose intracellular pathogens survival, macrophages modulate their iron uptake by inhibiting transferrin receptor1 (TfR1) expression. However, in case of visceral Leishmaniasis, *L. donovani* was found to reverse this mechanism by up-regulating TfR1 when labile iron pool in macrophages was depleted (31). Increased levels of TfR1 proteins serve to acquire easily transferrin-bound iron following host endocytosis and iron dissociation from transferrin by host endosomal acidic pH. In addition, the expression levels of TfR, and DMT1 were also increased in monocytes/macrophages from patients with post-kala-azar cutaneous leishmaniasis (32). Consequently, *Leishmania* is able to acquire transferrin-bound iron in the absence of its one transferrin uptake mechanism (33). Furthermore, the polarization into the M1 macrophages requires increased intracellular iron accumulation, while the transition to M2 macrophages is associated by decreasing iron sequestration and iron release (34). This suggests that the parasite may primarily exploit iron during the initial phase of macrophages activation rather than during the resorption phase.

Reports have shown the existence of species-specific adaptations in the regulation of heme acquisition through variations in the iron-dependent expression of the heme transporter (LHR1) in the context of cutaneous leishmaniasis (*L. amazonensis)* compared to visceral leishmaniasis *(L. infantum)* (35). Furthermore, *L. donovani* was shown to induce cleavage of the iron chaperones; poly(rC)-binding proteins (PCBPs). This cleavage mechanism blocks iron’s loading into ferritin, releases the label iron pool and increases the virulence factors of *Leishmania*, leading to its accumulation, growth and persistence within macrophages (36).

Overall, host iron metabolism is impacted by *Leishmania* infection and the host iron handling is deployed and/or bypassed by the parasite to ensure its virulence and survival.

## 3 Interplay between hepcidin and *Leishmania* infection

Hepcidin has been first discovered as cysteine-rich antimicrobial peptide that exhibited antifungal and antibacterial activities, and is mostly synthesized in the liver and excreted in urine (37, 38). Hepcidin gene (HAMP) encoded a prepropeptide composed of 84 amino acid containing a typical signal sequence and a consensus cleavage site for a prohormone convertase generating the bioactive 25–amino acid hepcidin (38, 39). Hepcidin, was then identified acts as hormone-like regulating negatively iron homeostasis (40, 41). Circulating hepcidin controls the iron efflux from duodenal enterocytes, and from the sequestering/recycling reticuloendothelial system; macrophages. In these later, hepcidin limits iron export by binding to FPN leading to the internalization and degradation of the iron exporter (42). However, in the absorptive cells, hepcidin inhibited FPN and additionally downregulates DMT1 importer (43).

The expression of hepcidin is predominantly regulated at the transcriptional level by various factors including iron level through BMP/SMAD signaling, erythropoiesis, hypoxia (12) and by inflammatory stimuli via JAK/STAT signaling, activated mainly by Interleukin (IL-6) (44–46). Several lymphoid and non-lymphoid cells including monocytes, T-lymphocytes, endothelial cells produce this pleiotropic cytokine (47). IL-6 and IL-6R interaction can activate both STAT1 and STAT3, but only STAT3 binds to hepcidin promoter (48, 49).

In instances of infection, hepcidin expression levels increase early, following IL-6 release by immune cells and Toll-like receptor (TLR) stimulation by Gram-negative bacteria-derived lipopolysaccharide (LPS). This elevation in hepcidin strengthens host defenses by acting on various barriers, particularly by restricting the availability of iron, which naturally prevents pathogen growth. However, other host defense mechanisms have also been shown to be induced by hepcidin, including the neutrophils recruitment, antibacterial activities, and environmental acidification in the gut and urinary tract (50–52). If hepcidin synthesis is upregulated in many bacterial infections, in the some viral infections, hepcidin may be increased or suppressed. Chronic hepatitis C virus (HCV) has been shown to suppress hepcidin and increase hepatic iron overload (53). However, some studies suggest that hepcidin is higher in severe cases of malaria, while others show unchanged or even reduced levels, making the understanding of its specific role challenging. Hepcidin levels upregulation correlated with a decrease in parasitemia, thereby iron limitation may facilitate infection control (54, 55). Nevertheless, a recent investigation revealed that the progression of the malaria hepatic stage was inhibited by interferons, but was independent to hepcidin suppression (56).

Additionally, extrahepatic hepcidin production has been identified in several tissues, particularly in epithelial barriers that are confronted with infections from external pathogens (23). The combined actions of hepatic and extrahepatic hepcidins likely have synergistic effects that help to prevent pathogen intrusion and invasion. Nonetheless, further research is necessary to explore how these hepcidins respond to pathogens that disseminate intracellularly, as in the case of *Leishmania.* The research in this area is ongoing, and further studies are needed to fully elucidate the interplay between hepcidin, iron metabolism, and *Leishmania* infection. *L. amazonensis* inducing skin lesions, seems to increase hepcidin expression in the liver of 9-week-old infected mice (57). Given that CL is mainly induced by *L. tropica* and *L. major* and exhibited a clinical polymorphism in Morocco, we confirmed that at least infection of *L. tropica* was associated with increased hepcidin in liver in 13 weeks-post infected Swiss mice (data not shown). Another study on *Leishmania* infection was conducted by Charlebois et al. (58) using a mouse model of hemochromatosis; hemojuvelin-deficient (Hjv KO) mice. Hemojuvelin is a cofactor in the bone morphogenetic protein (BMP)/SMAD signaling pathway, which plays a crucial role in stimulating hepcidin production. The study demonstrated that Hjv KO mice exhibited transient protection against cutaneous *L. major* infection but failed to show protection against visceral leishmaniasis caused by *L. infantum*. These data suggests that the cutaneous microenvironment may differ from other sites of infection in terms of cellular targets, iron availability, and possibly the role of hepcidin-induced local defenses.

Indeed, an *in vitro* study, showed that direct incubation of macrophages J774 cell line by exogenous hepcidin inhibits the *L. major* promastigotes growth via degradation of the iron exporter FPN and therefore an increase of toxic cytosolic iron pool (59). Another study examined Hepcidin/Nramp1(Natural Résistance Associated Macrophage Protein 1) axis in *L. major*–infected murine macrophages including J774 cell line. The results revealed that *L. major* induced local hepcidin in macrophages, which in turn induced Nramp1 downregulation (60). Nramp1 is expressed within phagolysosomal membrane and exports iron from phagocytic vesicles to cytosol in order to reduce susceptibility to infection with intracellular microbes including *Leishmania* (61, 33). These studies suggest that hepcidin can serve simultaneously as a virulence factor for *L. major* and as a defense mechanism for the host. Interestingly, an alternative study by Das et al. demonstrated that *L. donovani* reduces the abundance of ferroportin (FPN) to facilitate its growth within host macrophages through mechanisms that are independent of hepcidin (62). This finding further highlights the differences between cutaneous and visceral leishmaniasis in the regard of iron and hepcidin influences.

## 4 Impact of Iron loading and deprivation on *Leishmania* infection

Iron supplements were provided to mice, resulting in a reduction of parasitic load and the restoration of iron balance, restricting the development of cutaneous as well as visceral leishmaniasis. Indeed, it has been reported that iron loading confers a defense against *L. major* infection through the excessive oxidative burst production. This protective effect was also associated with NF-κB activation, promoting differentiation of CD4+ T cells into Th1 that induce M1 differentiation; which is crucial for limiting *Leishmania* growth (63–67). However, other therapeutic approaches are based on chelation of iron by reducing its availability in the host. In fact, *in vivo* studies have been carried out in a mouse model showing that ribonucleotide reductase and mitochondria act as a target when using quercetin and 2,2-Dipyridyl respectively. These components can act as iron chelators, with antileishmanial potential (68). Further research on the role of iron metabolism, during *Leishmania* infections, is needed to fully elucidate the mechanisms involved and their implications for treatment strategies.

## 5 Conclusion

Iron is vital to the parasite’s survival, but its accumulation in the cells requires meticulous control. This is ensured by maintaining a delicate balance, which is a strategic approach employed by the *Leishmania* parasite to avoid iron-induced damage. Therefore, attention to iron, through its regulation, could serve as a therapeutic strategy or medical goal for managing this infectious disease. The fluctuation in hepcidin level may indicate the parasite’s exploitation of iron metabolism, causing its disruption. Indeed, our understanding of how this parasite affects iron equilibrium via hepcidin is still limited and poorly investigated. In particular, the mechanism by which *Leishmania* infection induces an increase or decrease in hepcidin and its impact on the outcome of this infection still unclear. Hence, we are currently conducting research in this field, with experiments underway to address this issue and contribute to the advancement of therapeutic strategies in the fight against this parasitic infection.
